# Structural Heart Interventional Imagers - The New Face of Cardiac Imaging

**DOI:** 10.5935/abc.20180232

**Published:** 2018-11

**Authors:** João L. Cavalcante, Dee Dee Wang

**Affiliations:** 1 Minneapolis Heart Institute, Abbott Northwestern Hospital, Minneapolis - Minnesota - USA; 2 Valve Science Center, Minneapolis Heart Institute Foundation, Minneapolis, Minnesota - USA; 3 Center for Structural Heart Disease, Henry Ford Health System, Detroit - Michigan - USA

**Keywords:** Cardiology/education, Cardiology/trends, Diffusion of Innovation, Education, Medical, Graduate/trends, Multimodal Imaging/trends, Transcatheter Aortic Valve Replacement/economic.

With the aging of the world’s population, there has been a parallel growth of valvular heart disease. The development and establishment of less-invasive transcatheter aortic valve replacement (TAVR) has provided a different framework to approach these patients through a multi-disciplinary heart team for planning and treatment. This multi-disciplinary heart team allows the sharing of different expertise and knowledge in order to improve patient care. Although TAVR is one example, many other transcatheter structural heart interventions for the mitral valve, left atrial appendage, paravalvular leak closure, and tricuspid valve, will continue to expand the armamentarium of less-invasive therapies for these typically high-risk patients.

Within this context of continued expansion of devices and procedures, there has been increased demand for physicians with specific procedural-based skills and advanced cardiac imaging training in both echocardiography and cardiac computed tomography (CCT). However, the relative novelty of this subspecialty, brings many challenges. In the presence of poorly defined training requisites and skill-sets and lack of appropriate procedural reimbursement and recognition of the advanced level of peri-procedural imaging and medical care provided, there are many barriers to sustainability and expansion of this unique subspecialty.

## Training in structural heart disease imaging

Although training in multi-modality imaging has been well outlined,^1^ there are no specific training guidelines and/or requirements for SHD imagers as demonstrated by the results of a recent European survey.^2^ Some of the challenges currently faced by cardiology fellows who look for SHD imaging training include finding training centers with enough high-risk clinical volume and exposure to a variety of high-risk procedures so they can train beyond traditional TAVR procedures. This brings an inevitable question of whether adequate SHD imaging training should therefore be reserved to a small number of centers with sufficient knowledge and experience in these procedures. What should constitute the minimal portfolio of procedures, their degree of complexity, the number of cases performed for procedural planning and for intraprocedural guidance to achieve adequate proficiency are some of the questions whose answers remain unclear.

The majority of high-volume programs can provide comprehensive exposure for adequate training, particularly in TAVR, Atrial Septal Defect (ASD) and Left Atrial Appendage (LAA) closure procedures. Transcatheter mitral valve repair with MitraClip system (Abbott Vascular, Menlo Park, CA) is also becoming increasingly more commonly performed and should become a standard part of the SHD imager training. On the other hand, transcatheter procedures such as paravalvular leak closure, transcatheter mitral valve replacement and percutaneous tricuspid interventions are more complex and less frequently performed, and therefore should involve different expectations for what is considered the minimal requirement to achieve proficiency.

## Important job attributes

We have recently provided a brief outline including some of the main characteristics and attributes necessary for the success of Structural Heart Disease (SHD) imagers.^3^ One of the key components is to have exquisite understanding of and training in these imaging modalities so the imager can integrate and succinctly present information to the heart team, as well as provide value for further recommendations in diagnostic testing and interpretation of data, particularly when there are conflicting reports.

In pre-procedural planning, review and synthesis of serial imaging studies is required to evaluate for progressive changes in cardiac function, chamber size, and severity of valvular pathology. This is particularly important when multi-valvular disease is present, which can pose a challenge in both diagnostic and therapeutic decisions. More often than not, using multi-modality imaging and hemodynamic evaluation can be necessary to clarify the clinical question(s).

During intraprocedural guidance, SHD imagers learn to be agile, focused, mindful and able to protect themselves from radiation exposure. The ability to apply multi-modality critical thinking to integrate and combine clinical information and imaging findings (fluoroscopy and TEE) implies a physician trained skill-set that imagers can develop overtime. Interventional imaging physician driving critical-thinking imaging becomes invaluable to procedural success, much more than any form of imaging overlay or fusion. In-depth knowledge of particular devices and procedural steps, as well as clear, succinct and timely communication with the interventional cardiologist and other team members are critical attributes of a successful SHD Imager, thus implying solid knowledge of the timing and importance of his/her role.

Post-procedurally, SHD imagers must be able to correlate imaging findings with intraprocedural results and potential device complications. Exposure to a variety of SHD interventions is required in order to generate sufficient imaging experience, to allow the mitigation of complications and to promote safety during high-risk transcatheter procedures. A SHD imager who has developed these unique skill sets will be an indispensable asset to a SHD heart team and a key component to achieve excellent procedure safety and outcomes.

Given the dynamic nature of this field, continued changes are expected on the standard training curriculum, reflecting important updates in the medical literature, device iterations and procedural changes. This can be done by attending annual meetings and industry-sponsored seminars, participating in online CME opportunities and structural imaging workshops, all of which can help refresh and enhance imaging skills.

## Radiation exposure is a potential job hazard for the shd imager

Although the issue of radiation exposure was not adequately studied until relatively recently,^4^^,^^5^ it certainly represents one of the most important job hazards for the SHD imager. Both publications^4^^,^^5^ confirm that the SHD imager can be subject to very high levels of radiation exposure in structural cases.

Therefore, given the increased complexity of these procedures, which demand more fluoroscopic and imaging guidance, one can only hope that it remains an important area for future research and technological development. At present time, a number of simple measures, such as the use of protective lead apron, portable ceiling-suspended lead shield and distancing from the X-ray source, can provide important strategies to minimize exposure and the potential risk associated with it.^5^^,^^6^

Work environments and hospital management teams need to be supportive of and accomodating to providing the necessary resources that can minimize the potential consequences of excessive radiation exposure outlined by the authors.

## Reimbursement and sustainability of work enviroment

At the majority of programs in the United States, the SHD Interventional Imager is considered part of the non-invasive general cardiologist group. This occurs at private-practice groups, hospital-employed group-practices or at major academic centers. This creates a significant mismatch between the amount of time that is required to plan and guide complex SHD procedures, and the reimbursement currently allocated to the SHD imager. In the current model, the amount of work relative value units (wRVU) dictates the metrics for purposes of reimbursement and final wages. Simply put, the more procedures a physician does, the more studies he/she reads, the more he/she can charge.

The current model does not reflect the time spent on procedural planning, the required skill-set to successfully guide complex SHD interventions, nor does it account for the potential adverse health-effects on the SHD imager, such as radiation exposure. Let’s take, for example, an uncomplicated MitraClip procedure. This Mitraclip procedure is dependent on intraprocedural transesophageal (TEE) guidance, and requires 90+ mins of uninterrupted real-time TEE 3D imaging procedural guidance. This is billed as one umbrella SHD intraprocedural TEE code (93355), with an associated wRVU measure of 4.66, therefore amounting to a $230 charge. Within the same time frame, another “non-invasive” cardiologist could have read 10-15 transthoracic echocardiograms (valued at 1.3 wRVU per study) or 3-4 TEEs (valued at 2.3 wRVU per study), which demonstrates, by traditional productivity metrics, more value to an institution than the Interventional Imaging physician functioning as a second operator in the Mitraclip procedure who is additionally getting radiation exposure. [source: http://asecho.org/2018-medicare-physician-fee-schedule-final-rule].

SHD imagers must continue to advocate recognition for the unique requirements to thrive in this emerging subspecialty. Sustainability within a SHD imaging career track is directly dependent upon fair productivity metrics. Many graduating fellows show a clear interest in pursuing further SHD Interventional Imaging training. However, current reimbursement practice models will deter potential trainees from embracing this new subspecialty field of medicine. A salary-based model is more likely to facilitate a successful SHD imaging career, as opposed to the traditional wRVU productivity model. Until societal guidelines are established for this emerging field, differential procedural codes will continue to fall short on allocating and compensating SHD imaging time properly.

## Future directions

The presence of a skilled SHD imager is critical to the growth and success of any high-volume SHD program. The recent, strongly positive results of the COAPT trial^7^ emphasize the opportunity for a multi-societal level discussion. In order to allow sustainable growth and continue to provide the imaging support necessary for patient safety and the success of these high-risk transcatheter procedures, it is necessary to revise the current structural and payment model which provides insignificant acknowledgement to the SHD imager; a Co-operator who is absolutely necessary to successfully execute this procedure.

Together, these findings emphasize the critical need for and the opportunity to recognize SHD interventional imaging as a subspecialty within Cardiology and Cardiac Imaging and importantly, to legitimize the SHD imager as the second procedure operator, equally dedicated to exceptional patient care.
